# Altered Urinary Metabolomics in Hereditary Angioedema

**DOI:** 10.3390/metabo12111140

**Published:** 2022-11-19

**Authors:** Xue Wang, Yuxiang Zhi

**Affiliations:** Department of Allergy & Clinical Immunology, Peking Union Medical College Hospital, Peking Union Medical College, Chinese Academy of Medical Sciences, National Clinical Research Center for Immunologic Diseases, #1 Shuaifuyuan, Wangfujing, Beijing 100730, China

**Keywords:** hereditary angioedema (HAE), C1-inhibitor, metabolomics, mass spectrometry, pathogenesis

## Abstract

Hereditary angioedema (HAE) is a rare and potentially life-threatening disease with heterogeneous clinical symptoms. The metabolomic profile of HAE remains unknown. Uncovering the metabolic signatures of HAE may provide inspiration for a comprehensive understanding of HAE pathogenesis and may help explore potential new metabolic biomarkers. We performed a comprehensive metabolic analysis using high-performance liquid chromatography–tandem mass spectrometry (HPLC-MS/MS). Urine samples from 34 HAE patients and 82 healthy controls (HCs) were collected to characterize the metabolic signatures associated with HAE. The metabolomes of HAE patients carrying different mutation types were also compared. A total of 795 metabolites were accurately detected and quantified. We considered 73 metabolites as differential metabolites in HAE patients (with an importance in projection (VIP) value > 1.0, *q*-value < 0.05, and fold change (FC) ≥ 1.2 or FC ≤ 0.8). Several metabolites associated with riboflavin metabolism, the citrate cycle, oxidative stress, and inflammation, including xanthine, oxypurinol, vitamin B2, and isocitrate, were significantly altered in HAE patients. No significantly different metabolites were found in HAE patients carrying different mutation types. The present study highlights that metabolic disturbances in the purine metabolism, riboflavin metabolism, and TCA cycle may be involved in the pathogenesis of HAE. Although biochemical significance requires further experimental verification, these findings may help to identify novel candidate metabolite biomarkers associated with HAE.

## 1. Introduction

Hereditary angioedema (HAE, OMIM #106100) is an autosomal dominant disease with a worldwide prevalence of 1/50,000 [1]. HAE presents with recurrent, unpredictable episodes of cutaneous and submucosal edema that typically affects the face, extremities, trunk, gastrointestinal tract, and upper respiratory tract [2]. Gastrointestinal edema can present as severe abdominal pain and is often misdiagnosed as an acute abdomen, which leads to unnecessary surgery [1]. Laryngeal edema is the most dangerous symptom, resulting in a 40% lifetime mortality rate [1].

Common forms of HAE include C1 inhibitor (C1-INH) deficiency (HAE-1) and C1-INH dysfunction (HAE-2), both of which are caused by variants in the *SERPING1* gene [2]. Currently, about 900 different variants in the *SERPING1* gene have been reported to be associated with HAE-1/2 [3]. In HAE-1/2, functional C1-INH is deficient, and, consequently, the kallikrein-kinin system is overactivated, thereby, leading to the overproduction of bradykinin [4]. Bradykinin can bind to bradykinin B2 receptors, thereby, increasing vascular permeability and leading to angioedema attacks [4]. In recent years, other forms of HAE have been reported. These HAE patients have normal C1-INH levels, and some of them have mutations in factor XII [5], angiopoietin-1 [6], plasminogen [7], kininogen [8], myoferlin [9], or heparan sulfate-glucosamine 3-O-sulfotransferase 6 [10].

Recently, several other pathways have been proposed to have potential correlation with HAE, including inflammation and oxidative stress [11,12]. However, the underlying molecular mechanism is unclear. Metabolomics represents a “functional readout of the physiological state” [13] and is increasingly used to identify novel biomarkers in several fields, including some hereditary disorders [14,15], neurodegenerative diseases [16], and cardiovascular diseases [17]. Therefore, the purpose of this study is to identify the metabolomic profiles of HAE patients and to investigate the differences in the metabolic profiles of HAE patients with different mutation types. This assessment may help to further characterize the molecular pathology of HAE and to identify novel candidate metabolite biomarkers associated with HAE.

## 2. Materials and Methods

### 2.1. Study Participants

A total of 34 drug-naïve HAE patients and 82 healthy controls (HCs) were recruited at the Peking Union Medical College Hospital from November 2020 to September 2021. 

The diagnosis of HAE was made according to the typical clinical presentation (recurrent skin swelling, gastrointestinal attacks, and/or laryngeal edema) and repeated laboratory tests with abnormal C1-INH function, C1-INH protein, and C4 [2]. The disease severity was assessed with a visual analogue scale [18]. To exclude the potential impact of treatment on metabolomics, only patients who never received any of the on-demand treatment, short-term prophylaxis, or long-term prophylaxis were included. HCs without edema-associated disease, severe metabolic diseases, autoimmune diseases, or cancer were recruited from health examination center. This study was approved by the Research Ethical Committee of the Peking Union Medical College Hospital (Number: I-22PJ198). Each study subject signed a written informed consent.

### 2.2. Sample Collection

Midstream urine samples were collected in the morning before breakfast for HAE patients and HCs. For HAE patients, urine samples were collected during the remission period (≥2 weeks without an episode). A total of 10 mL urine was obtained from each participant and immediately stored at −80 °C. 

Peripheral blood from HAE patients was obtained with EDTA blood collection tubes and stored at −80 °C until genomic DNA was isolated.

### 2.3. Metabolite Extraction

Urine metabolite extraction was performed based on the method of J Want et al. [19] and Barri et al. [20]. Urine samples (100 μL) were placed in Eppendorf tubes and resuspended by well vortex with prechilled 80% methanol. After, the samples were incubated on ice for 5 min and centrifuged at 15,000 *g* for 20 min at 4 °C. An amount of supernatant was diluted with mass spectrometry grade water to a methanol content of 53%. Next, the samples were transferred to a fresh Eppendorf tube and then centrifuged at 15,000 *g* at 4 °C for 20 min. Finally, the supernatant was injected into the HPLC-MS/MS system. To ensure analytical stability, quality control (QC) samples were prepared by mixing equal aliquots of all samples. Blank samples were made with 53% methanol solution instead of urine and treated with the same methods.

### 2.4. HPLC-MS/MS Analysis

High-performance liquid chromatography–tandem mass spectrometry (HPLC-MS/MS) analysis was performed at Novogene Co., Ltd. (Beijing, China). The ExionLCTM AD system (SCIEX) combined with a QTRAP^®^ 6500+ mass spectrometer (SCIEX) was used. Samples were injected onto an Xselect HSS T3 (2.1 × 150 mm, 2.5 μm) using a 20-min linear gradient at a flow rate of 0.4 mL/min for the positive/negative polarity mode. The eluents included A (0.1% formic acid–water) and B (0.1% formic acid–acetonitrile) [21]. 

The solvent gradient was set as the following: 2% B, 2 min; 2–100% B, 15.0 min; 100% B, 17.0 min; 100–2% B, 17.1 min; and 2% B, 20 min. In positive polarity mode, the QTRAP^®^ 6500+ mass spectrometer was operated with Curtain Gas at 35 psi, Collision Gas at medium, IonSpray Voltage at 5500 V, temperature at 550 °C, Ion Source Gas at 1:60, and Ion Source Gas at 2:60. In negative polarity mode, the QTRAP^®^ 6500+ mass spectrometer was operated with Curtain Gas at 35 psi, Collision Gas at medium, IonSpray Voltage at −4500 V, temperature at 550 °C, Ion Source Gas at 1:60, and Ion Source Gas at 2:60.

### 2.5. Metabolite Identification and Quantification

Quasi-targeted metabolomics, a method for the semi-quantitative analysis of metabolites, was applied in this study. Based on the Novogene in-house database, the experimental samples were detected using MRM (Multiple Reaction Monitoring). The Q3 (daughter ion) was used for the semi-quantitative analysis of metabolites. The Q1 (parent ion), Q3, RT (retention time), DP (declustering potential), and CE (collision energy) were utilized to identify metabolites. The SCIEX OS Version 1.4 was applied to handle the data files derived from HPLC-MS/MS to integrate and correct the peak. The main parameters were set as the following: minimum peak height, 500; signal/noise ratio, 5; and Gaussian smooth width, 1. The area of each peak indicates the relative content of the corresponding substance. 

Metabolites with more than 50% missing values in the samples were excluded. To assess the variability from extraction, the relative standard deviation (RSD%) of the QC samples was calculated. Metabolites with RSD% > 50 in the QC samples were filtered.

### 2.6. Genetic Analysis

Genetic analysis was performed on HAE patients. The DNA extraction methods, polymerase chain reaction amplification and Sanger sequencing analysis methods were described in detail elsewhere [22]. 

### 2.7. Statistical Analysis

Metabolites were annotated with several databases, including the KEGG database (http://www.genome.jp/kegg/, accessed on 22 September 2020), HMDB database (http://www.hmdb.ca/, accessed on 23 February 2022), and Lipidmaps database (http://www.lipidmaps.org/, accessed on 5 November 2021). Univariate and multivariate statistical analyses were performed to assess the significance of each metabolite. 

Multivariate analysis was performed with the web-based application MetaboAnalyst 5.0 (https://www.metaboanalyst.ca/, accessed on 30 June 2022). After data scaling and normalization (normalization by sum, log transformation, and auto scaling), unsupervised principal component analysis (PCA) was performed to observe clustering. The supervised partial least squares-discriminant analysis (PLS-DA) was used to determine the discriminant metabolites implicated in metabolomics signatures. We applied 10-fold cross validation to validate the PLS-DA model. The performance of PLS-DA model was assessed using the R^2^ value and Q^2^ value. In the PLS-DA model, metabolites with an importance in projection (VIP) value > 1.0 were considered significant.

Univariate analysis (t-test) was performed with R software (R Foundation for Statistical Computing, version 3.5.0). A standard Benjamini–Hochberg approach was used to control the false discovery rate (FDR). A *q*-value (FDR corrected *p*-value) less than 0.05 was set as the cut-off value. Fold-change (FC) values were calculated as the ratio between the mean intensity values of the comparison groups. Finally, metabolites with VIP > 1.0, *q*-value < 0.05, and FC ≥ 1.2 or FC ≤ 0.8 were identified as differential metabolites.

## 3. Results

Table 1 shows the characteristics of the participants. The average age of HAE patients and HCs were 44.2 and 40.3 years, respectively. One HAE patient and three HCs had hypertension. The participants had no other chronic diseases, including diabetes, autoimmune diseases, and cancer. In HAE, 91.2%, 58.8%, and 70.6% of patients reported previous skin edema, gastrointestinal edema, and laryngeal edema, respectively. The median annual frequency of edema attacks was 5, and the mean disease severity score was 7.3.

A total of 795 metabolites were accurately detected and quantified. The most important classes included amino acids and amino acid derivatives (21.5%), organic acids and organic acid derivatives (16.6%), nucleotides and nucleotide derivates (9.2%), carbohydrates and carbohydrate derivatives (7.7%), and fatty acyls (7.3%). The remaining metabolites belonged to amines, benzene and substituted derivatives, benzoic acid and its derivatives, pyridine and its derivatives, cholines, bile acids, polyamine, hormones, phospholipid, pyrimidines and pyrimidine derivatives, purines and purine derivatives, carnitine, ketones, vitamins, eicosanoids, esters, and other families.

### 3.1. Metabolic Signatures of HAE Patients

**Figure 1:**
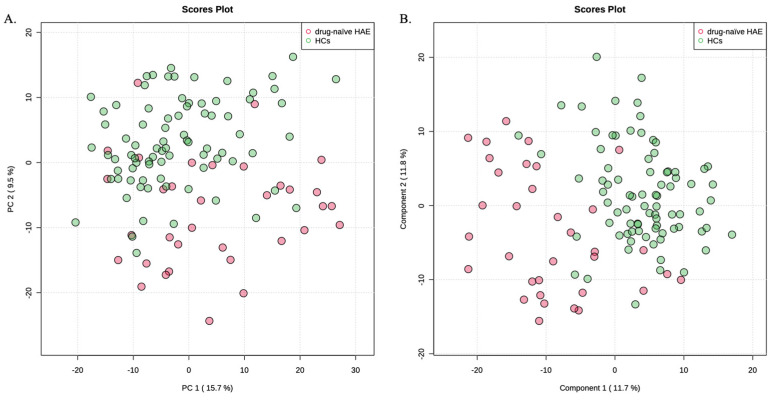
(**A**) PCA score plot of HAE patients and HCs. (**B**) PLS-DA score plot of HAE patients and HCs.

Drug-naïve HAE patients and HCs were compared to identify the metabolic signatures of drug-naïve HAE patients. This could exclude potential effects of treatment. The unsupervised PCA method revealed no outlier sample (Figure 1A). Therefore, all 34 HAE patients and 82 HCs were included in the subsequent data analyses. PLS-DA model was constructed to identify differential metabolites in HAE (Figure 1B). Cross-validation was conducted to make sure that the model was not over-fitted (R^2^ = 0.47, Q^2^ = 0.36). Of the 795 metabolites, 314 metabolites with VIP values > 1.0 contributed significantly to differentiating HAE patients from HCs. 

After univariate analysis with FDR calibration, 104 metabolites were significantly changed with a *q*-value < 0.05. In addition, 152 metabolites had FC < 0.8, and 122 metabolites had FC > 1.2. Finally, the overlapping 73 metabolites were identified as differential metabolites in HAE patients (Table 2). The molecular weights, Q1 m/z values, and adduct forms are shown in Table S1. Of these, 59 metabolites were significantly decreased in HAE patients, and 14 metabolites were significantly increased in HAE patients.

### 3.2. Metabolic Signatures of HAE Caused by a Premature Stop Codon

Next, the metabolic differences between different mutation types were investigated. Genetic analysis was performed on 25 of the 34 HAE patients. By Sanger sequencing, 20 of these patients were detected as carrying pathogenic variants. Table 3 demonstrates the clinical characteristics and genetic variants of these 20 patients. Two HAE-2 patients carrying the Arg^466^ variant were excluded from further metabolomics analysis. Therefore, 18 HAE-1 patients were included and grouped according to their mutation type: Group A, 6 patients carrying missense or in-frame variants; and Group B, 12 patients carrying nonsense or frameshift variants. Both nonsense and frameshift variants can form premature stop codons, which then trigger the rapid degradation of mRNA. 

PCA showed no outliers in 18 HAE-1 samples (Figure S1A). PLS-DA showed clear group separation (Figure S1B; R^2^ = 0.64, Q^2^ = 0.22). A total of 276 metabolites contributed significantly (VIP >1.0) to distinguish HAE caused by a premature stop codon mutation from HAE caused by a missense or in-frame mutation. However, no metabolites remained as significant after univariate analysis with FDR correction. We observed that some metabolites with VIP >1.0 were not significantly different in the univariate analysis, which may be due to these metabolites potentiating each other in the multivariate analysis.

## 4. Discussion

The pathophysiological mechanism involved in HAE-1/2 is the lack of functional C1-INH, which regulates a variety of proteases in the kallikrein-kinin system, complement, coagulation, and fibrinolytic pathways [4]. Bradykinin, produced through the activation of the kallikrein-kinin system, is thought to be the major mediator of HAE-1/2 [4]. In recent years, several other potential events involved in HAE have been reported, including inflammation and oxidative stress [11,12]. Several pro-inflammatory cytokines and oxidative stress markers have been found to be increased in HAE patients, suggesting complex changes in biochemical pathways in HAE patients. Metabolites are crucial mediators in biological systems and can indicate abnormalities in biochemical pathways [29]. To the best of our knowledge, this is the first study to explore metabolic changes in HAE patients. 

Our findings showed that the metabolomic signatures of HAE patients were substantially different from those of HCs. Based on a quasi-targeted metabolomics approach, 73 metabolites were found to be significantly altered in HAE patients. Some metabolites involved in the purine metabolism, riboflavin metabolism, TCA cycle, and some metabolites with anti-inflammatory and antioxidant effects were significantly altered in HAE patients.

Xanthine, hypoxanthine, oxypurinol, and uric acid were significantly reduced in HAE patients. Hypoxanthine produces xanthine under the catalysis of xanthine oxidase, which constitutes the purine metabolic pathway. [30]. Oxypurinol is a xanthine oxidase inhibitor [31]. Uric acid is the final oxidation product of the purine metabolism. Considering the alterations in these metabolites and, in particular, the decrease in the xanthine oxidase inhibitor oxypurinol, we speculate that the xanthine/xanthine oxidase system may be imbalanced. 

A previous study reported that the xanthine/xanthine oxidase system can selectively enhance bradykinin-induced vasodilation by generating low concentrations of ROS in the coronary circulation of isolated guinea pig hearts [32]. Bradykinin-induced vascular leakage is the fundamental abnormality in HAE-1/2 [2]. However, it is not yet clear whether purine metabolites and the xanthine/xanthine oxidase system will potentiate vascular leakage in HAE patients, and the potential impact needs to be studied in the future. 

Vitamin B2, or riboflavin, was decreased in HAE patients compared to HCs. Vitamin B2 plays an important role in the maintenance of human health, including antioxidant, anti-inflammatory, anti-aging, and anti-cancer [33]. Currently, no study has reported the association between vitamin B2 and HAE. Based on the role of vitamin B2 and the pathological mechanism of HAE, it is speculated that the antioxidant and anti-inflammatory properties of vitamin B2 may be associated with HAE. Experimental studies have shown that vitamin B2 improves the synthesis of the normal extracellular matrix and reduces the level of reactive oxygen species (ROS) [34]. 

Animal studies have shown that vitamin B2 reduces the risk of cardiac dysfunction in diabetic rats by improving heart oxidant status and increasing antioxidants [35]. Recent studies supported that oxidative stress plays an important role in the pathophysiological mechanisms of HAE. Del Giacco et al. demonstrated that circulating levels of oxidative stress markers, including advanced glycation end products and advanced oxidation protein products, were significantly elevated in HAE with C1-INH deficiency [36]. Obtulowicz et al. showed higher basal and hydrogen-peroxide-induced ROS levels in HAE compared to controls [37]. 

All the evidence mentioned above suggests that patients with HAE may have disturbed redox homeostasis and abnormal oxidative stress [37]. In addition to antioxidant stress, anti-inflammation is also an important property of vitamin B2. Vitamin B2 has been reported to reduce plasma pro-inflammatory cytokines, including tumor necrosis factor alpha (TNF-α), interleukin-1 beta (IL-1β), and nitric oxide (NO) [33,38,39]. Previous studies have shown that there is an inflammatory activation process in HAE [12]. During the onset and remission of HAE, some cytokines produced by T helper 17 were significantly higher than in controls [12]. 

IL-1 and TNF-α can enhance the activation of the prekallikrein–high-molecular-weight kininogen complex, which is the initiating event of an angioedema attack [40]. Inflammatory stimuli can also induce the expression of bradykinin B1 receptor [41], and reduced vitamin B2 may exacerbate this process. In addition, intraperitoneal administration of vitamin B2 has been reported to reduce vascular leakage in rats [42]. It is unclear whether reduced vitamin B2 exacerbates vascular leakage in HAE patients or whether it affects the course of HAE by inducing oxidative stress or inflammation, and the specific effects of vitamin B2 on HAE remain to be explored.

Disruption of the TCA cycle was observed in HAE patients. Several metabolites of the TCA cycle, including isocitrate and succinic acid, were significantly reduced in HAE patients. In addition, citric acid, also a metabolite in the TCA cycle, was decreased in HAE patients with FC < 0.8, VIP > 1.0, and *p*-value < 0.05, despite *q*-values > 0.05. The disruption of the TCA cycle in HAE patients suggests a deficiency of adenosine triphosphate (ATP) production in HAE patients. ATP was found to reduce the permeability of human umbilical vein endothelial cells [43]. Therefore, we hypothesize that impaired activity of the TCA cycle and subsequent ATP reduction may play a key role in HAE by increasing the vascular permeability.

Our results also showed that hydrocortisone (cortisol) and cortisone were decreased in HAE patients. Hydrocortisone protects the endothelial glycocalyx, maintains the vascular barrier, reduces interstitial edema [44,45], and inhibits immune and inflammatory responses [46]. Previous research reported that adrenocorticotropic hormone (ACTH) was significantly decreased in HAE-2 and was not affected by danazol treatment [47]. However, serum hydrocortisone levels were similar in healthy subjects, HAE-1 patients, and HAE-2 patients [47]. 

Compared with healthy women and female patients who did not take danazol, only female patients who had received danazol treatment had lower serum hydrocortisone levels [47]. In the present study, hydrocortisone levels were decreased in HAE patients compared with HCs. Our results were not fully consistent with previous research, which might be due to the different sex ratios of the included patients. In addition, other metabolites, including quinone, N-methylhydantoin, 2-aminooctanoic acid, etc., were also significantly altered in patients with HAE. There is no evidence for a biological explanation, and the function of these metabolites still needs to be further explored.

We further investigated the metabolic differences between HAE caused by premature stop codon mutations in the *SERPING1* gene and HAE caused by missense or in-frame variant. In this study, 12 patients carried nonsense or frameshift variants in the *SERPING1* gene. This would lead to a defect in C1-INH synthesis through nonsense-mediated mRNA decay [48]. We identified 11 nonsense or frameshift variants in these 12 patients, including c.172_181del, c.197dup, c.635dup, c.733_736dup, c.748_749del, c.897del, c.941_942insTC, c.944del, c.1051del, c.1106del, and c.1480C>T. These variants create premature stop codons at the 58th, 67th, 213th, 246th, 250th, 299th, 315th, 315th, 351st, 369th, and 494th amino acid sites, respectively. 

Except for c.1480C>T, all premature stop codons are located upstream of the reaction key, Arg^466^. This is a key residue of C1-INH that binds to the active site of the target protease. As a result, in patients with HAE caused by premature stop codon mutations, the synthesized protein will lack the reaction loop and will not recognize the target protease [49]. However, no metabolite was considered significantly changed in patients with different mutation types. This may be because the mutation types in the *SERPING1* gene were not significantly associated with the phenotype or severity of HAE patients (21, 33). Therefore, HAE patients with different mutation types may have a similar metabolic profile. In addition, smaller sample sizes may also lead to insignificant changes in the metabolite levels between groups.

The study has several limitations. First, due to the rarity of HAE, the study was conducted with a limited sample size. The potential differential metabolites identified in our study should be validated in a multicenter study with a larger sample size. Second, due to the unpredictability of HAE edema episodes, we only collected samples from patients in the remission period. However, the metabolomic profiles during an edema episode could be more informative for the pathogenesis of HAE. Future studies should explore the metabolomic changes during edema episodes in HAE.

## 5. Conclusions

In conclusion, this research emphasizes that metabolic disturbances in the purine metabolism, riboflavin metabolism, and TCA cycle are important metabolic events in HAE. The evaluations of metabolic changes could contribute to our understanding of HAE pathogenesis and promote the screening of diagnostic biomarkers. The differential metabolites identified in this study are statistically significant but not necessarily biochemically significant. Therefore, multicenter, large sample studies are needed to validate our results. 

## Figures and Tables

**Table 1:** Characteristics of HAE patients and healthy controls.

	Drug-Naïve HAE	Healthy Controls
n	34	82
Female	25 (73.5)	54 (65.9)
Male	9 (26.5)	28 (34.1)
Age (y)	44.2 ± 10.7	40.3 ± 10.5
Hypertension	1 (2.9)	3 (3.6)
Skin edema	31 (91.2)	NA
Gastrointestinal edema	20 (58.8)	NA
Laryngeal edema	24 (70.6)	NA
Annual attack frequency	5 [1,2,3,4,5,6,7,8,9,10,11,12,13,14]	NA
Disease severity	7.3 ± 1.5	NA

Data are presented as the mean ± SD, median [IQR], or n (%).

**Table 2:** The 73 most discriminant metabolites in HAE patients.

Metabolite	Class	HMDB ID	QC, RSD%	FC	*p*-Value	*q*-Value	VIP Value
Quinone	Ketones	HMDB0003364	33.37	2.65	0.0000	0.0008	2.21
N-Methylhydantoin	Organoheterocyclic compounds	HMDB0003646	10.51	1.2	0.0002	0.0042	2.20
2-Aminooctanoic acid	Organic acid	HMDB0000991	9.65	0.4	0.0000	0.0001	2.12
2,6-Dimethoxybenzoic acid	Benzoic acid	HMDB0029273	15.03	1.83	0.0015	0.0178	2.11
Thymine	Nucleotide	HMDB0000262	25.15	2.37	0.0000	0.0013	2.10
Propionylcholine	Cholines	HMDB0013305	11.72	0.41	0.0000	0.0001	2.08
1-Methylxanthine	Purines and purine derivatives	HMDB0010738	4.28	0.27	0.0000	0.0000	2.04
D-Glyceraldehyde 3-phosphate	Organic acid	HMDB0001112	8.98	1.79	0.0029	0.0298	2.00
O-Acetyl-L-carnitine	Carnitine	HMDB0000201	9.58	0.33	0.0000	0.0008	2.00
Hypoxanthine	Nucleotide	HMDB0000157	8.52	0.53	0.0000	0.0013	1.95
Acetylcarnitine	Carnitine	HMDB0000201	7.71	0.36	0.0000	0.0009	1.94
Trehalose 6-phosphate	Carbohydrates	HMDB0001124	10.28	1.95	0.0047	0.0417	1.90
1,4-Dihydro-1-Methyl-4-Oxo-3-Pyridinecarboxamide	Pyridine	HMDB0004194	3.28	0.55	0.0000	0.0011	1.87
2,4-Dihydroxybenzoic Acid	Benzoic acid	HMDB0029666	17.12	0.54	0.0000	0.0008	1.86
L-Octanoylcarnitine	Carnitine	HMDB0000791	3.12	0.44	0.0000	0.0009	1.86
Oxypurinol	Nucleotide	HMDB0000786	4.18	0.54	0.0000	0.0011	1.86
DL-Citrulline	Amino acid	HMDB0000904	5.15	2	0.0054	0.0440	1.85
Carnitine-C8	Carnitine	NA	3.16	0.44	0.0000	0.0009	1.84
Xanthine	Nucleotide	HMDB0000292	3.69	0.56	0.0000	0.0013	1.81
L-Alanine	Amino acid	HMDB0000161	5.19	1.38	0.0053	0.0440	1.78
Adrenochrome	Organoheterocyclic compounds	HMDB0012884	2.91	0.44	0.0000	0.0008	1.77
Hippuric acid	Organic acid	HMDB0000714	2.59	0.44	0.0000	0.0008	1.77
Taurochenodeoxycholic acid	Bile acids	NA	6.20	0.55	0.0001	0.0017	1.77
D-3-Phenyllactic acid	Organic acid	HMDB0000563	3.43	1.76	0.0053	0.0440	1.75
Glucarate O-Phosphoric Acid	Carbohydrates	NA	23.71	1.48	0.0064	0.0495	1.70
Vitamin B2	Vitamins	HMDB0000244	8.20	0.35	0.0000	0.0013	1.70
Hexanoylcarnitine	Carnitine	HMDB0000705	3.83	0.55	0.0000	0.0013	1.69
L-Homocystine	Amino acid	HMDB0000676	25.03	0.64	0.0000	0.0011	1.69
Carnitine-C6	Carnitine	NA	4.40	0.55	0.0001	0.0016	1.67
Decanoylcarnitine	Carnitine	HMDB0000651	5.00	0.52	0.0001	0.0016	1.67
Dodecanoylcarnitine	Carnitine	HMDB0002250	3.92	0.55	0.0003	0.0050	1.67
Carnitine-C12	Carnitine	NA	4.96	0.55	0.0003	0.0050	1.65
Isovalerylcarnitine	Carnitine	HMDB0000688	3.59	0.55	0.0001	0.0031	1.65
5-Aminosalicylate	Organic acid	HMDB0014389	22.26	0.56	0.0001	0.0017	1.64
Carnitine-C3	Carnitine	NA	3.50	0.49	0.0004	0.0063	1.63
Carnitine-C5	Carnitine	NA	3.47	0.55	0.0001	0.0034	1.63
Hydrocortisone	Hormones	HMDB0000063	4.42	0.44	0.0001	0.0016	1.62
2-Methylbutyroylcarnitine	Fatty acyls	HMDB0000378	2.46	0.56	0.0002	0.0035	1.61
Cholate	Bile acids	NA	7.07	2.74	0.0053	0.0440	1.61
N-Propionylglycine	Amino acid	HMDB0000783	10.05	1.35	0.0057	0.0450	1.57
3-Oxo-7alpha,12alpha-hydroxy-5beta-cholanoic acid	Bile acids	NA	18.39	1.67	0.0047	0.0417	1.56
Cinnamoylglycine	Amino acid	HMDB0011621	4.36	0.57	0.0002	0.0042	1.56
O-Acetyl-L-homoserine	Amino acid	HMDB0029423	4.55	0.49	0.0001	0.0029	1.55
L-Carnitine	Carnitine	HMDB0000062	5.97	0.52	0.0002	0.0042	1.52
Propionyl-L-carnitine	Carnitine	HMDB0000824	4.58	0.5	0.0003	0.0050	1.52
L-Alanyl-L-Lysine	Amino acid	HMDB0028692	6.47	0.52	0.0003	0.0056	1.49
Cortisone	Others	HMDB0002802	4.79	0.62	0.0002	0.0042	1.43
Methylmalonate	Organic acid	HMDB0000202	4.56	0.59	0.0008	0.0110	1.43
3-Hydroxy-3-Methylpentane-1,5-Dioic acid	Fatty acyls	NA	7.25	0.52	0.0003	0.0051	1.39
DL-Norepinephrine	Hormones	NA	39.22	0.57	0.0005	0.0072	1.34
Succinic acid	TCA cycle	HMDB0000254	2.83	0.61	0.0011	0.0143	1.34
Nonanoic acid	Fatty acyls	HMDB0000847	21.30	1.24	0.0055	0.0443	1.33
3-OH-anthranilate	Organic acid	NA	16.94	0.59	0.0000	0.0013	1.31
Deoxyguanosine	Nucleotide	HMDB0000085	9.15	0.65	0.0002	0.0042	1.31
Uric acid	Organic acid	HMDB0000289	4.89	0.66	0.0001	0.0016	1.30
Pantetheine	Amino acid	HMDB0003426	11.55	0.63	0.0005	0.0072	1.27
Guanethidine	Organic acid	NA	44.51	0.49	0.0000	0.0008	1.25
D-Glucosamine 6-phosphate	Sugar acids	HMDB0001254	16.26	0.49	0.0004	0.0057	1.16
Indoxylsulfuric acid	Organic acid	HMDB0000682	4.06	0.69	0.0028	0.0297	1.16
CMPF	Fatty acyls	HMDB0061112	5.03	0.57	0.0019	0.0218	1.14
Hydroquinone	Phenols	HMDB0002434	4.92	0.6	0.0009	0.0121	1.12
p-cresol	Phenols	HMDB0001858	5.38	0.69	0.0054	0.0440	1.11
3-Hydroxy-butyryl carnitine	Carnitine	NA	6.95	0.3	0.0010	0.0132	1.10
Isocitrate	Organic acid	HMDB0001874	11.40	0.67	0.0040	0.0375	1.09
Tauroursodeoxycholic acid Dihydrate	Bile acids	NA	7.16	0.48	0.0036	0.0339	1.09
4-Aminobenzoate	Benzoic acid	HMDB0004992	17.89	0.61	0.0005	0.0070	1.08
Tauro-alpha-Muricholic acid	Bile acids	NA	34.87	0.54	0.0016	0.0190	1.08
Taurocholic acid	Bile acids	HMDB0000036	7.35	0.6	0.0008	0.0116	1.08
Furfural	Aldehydes	HMDB0032914	27.54	0.64	0.0001	0.0017	1.06
O-Anisic Acid	Benzoic acid	HMDB0032604	5.27	0.62	0.0020	0.0220	1.05
Biotin	Vitamins	HMDB0000030	7.32	0.67	0.0048	0.0419	1.03
L-Glutamine O-Hexside	Amino acid	NA	11.32	0.57	0.0023	0.0250	1.03
Pyridoxamine	Pyridine	HMDB0001431	16.36	0.7	0.0014	0.0173	1.01

Abbreviations: HMDB, Human Metabolomics Data Base; QC, quality control, RSD%, percentage relative standard deviation.

**Table 3:** Characteristics of 20 HAE patients carrying pathogenic variants.

Group	Sex	Age (y)	DNA Change	Protein	HAE Type	References
A	F	40	c.1A>G	p.(Met1Val)	1	[23]
A	F	45	c.816_818del	p.(Asn272del)	1	[3]
A	F	32	c.1223A>G	p.(Asp408Gly)	1	[24]
A	M	28	c.1289T>C	p.(Leu430Pro)	1	[25]
A	M	28	c.1289T>C	p.(Leu430Pro)	1	[25]
A	M	31	c.1289T>G	p.(Leu430Arg)	1	[24]
B	F	51	c.172_181del	p.(Pro58Argfs * 18)	1	[24]
B	F	47	c.197dup	p.(Thr67Aspfs * 15)	1	[24]
B	F	56	c.635dup	p.(Phe213Leufs * 44)	1	[24]
B	F	45	c.733_736dup	p.(Ser246Lysfs * 12)	1	[24]
B	F	46	c.748_749del	p.(Val250Profs * 6)	1	VCV001329454.1
B	M	29	c.897del	p.(Trp299 *)	1	This study
B	F	45	c.941_942insTC	p.(Phe315Profs * 7)	1	[24]
B	F	40	c.944del	p.(Phe315Serfs * 6)	1	[3]
B	M	44	c.1051del	p.(His351Thrfs * 3)	1	[24]
B	F	47	c.1051del	p.(His351Thrfs * 3)	1	[24]
B	F	49	c.1106del	p.(Asp369Alafs * 28)	1	[26]
B	M	47	c.1480C>T	p.(Arg494 *)	1	[27]
-	F	48	c.1396C>A	p.(Arg466Ser)	2	[28]
-	F	49	c.1396C>A	p.(Arg466Ser)	2	[28]

Abbreviations: F, female; M, male. The * stands for termination codon in genetics.

## Data Availability

The data presented in this study are available on request from the corresponding author. The data are not publicly available due to privacy concerns.

## Supplementary Materials

The following supporting information can be downloaded at: https://www.mdpi.com/article/10.3390/metabo12111140/s1, Figure S1: (A) Unsupervised PCA score plot of HAE caused by a missense or in-frame mutation (Group A) and HAE caused by a premature stop codon mutation (Group B); (B) Supervised PLS-DA score plot of HAE caused by a missense or in-frame mutation (Group A) and HAE caused by a premature stop codon mutation (Group B). Table S1. Molecular weights, Q1 m/z values, and adduct forms of metabolites.
