# Supplementary material for: Altered Urinary Metabolomics in Hereditary Angioedema
Source: Metabolites. 2022 Nov 19;12(11):1140. doi: 10.3390/metabo12111140 (PMC9696332; doi:10.3390/metabo12111140)
Supplement: Supplementary file 1 [file metabolites-12-01140-s001.zip › metabolites-1994699-supplementary.pdf]

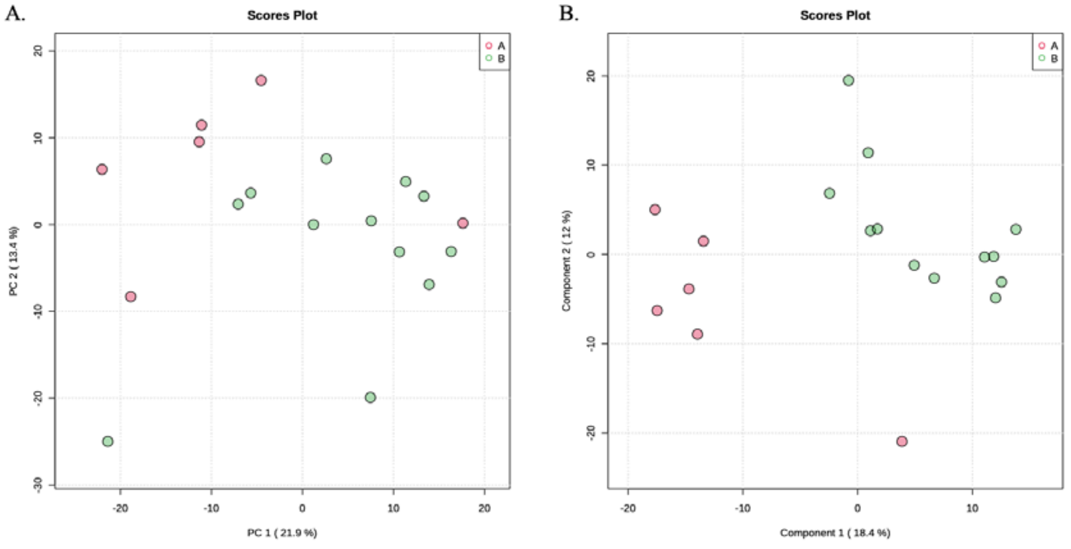

Figure S1. (A) Unsupervised PCA score plot of HAE caused by a missense or in-frame mutation (Group A) and HAE caused by a premature stop codon mutation (Group B); (B) Supervised PLS-DA score plot of HAE caused by a missense or in-frame mutation (Group A) and HAE caused by a premature stop codon mutation (Group B).

Table S1. Molecular weights, Q1 m/z values, and adduct forms of metabolites

Metabolite	Molecular Weight	Q1 m/z	Adduct form
Quinone	108.09	109	M+H
N-Methylhydantoin	114.1	113	M-H
2-Aminooctanoic acid	159.226	160	M+H
2,6-Dimethoxybenzoic acid	182.17	183	M+H
Thymine	126.113	127.1	M+H
Propionylcholine	160.234	160	M+H
1-Methylxanthine	166.14	167.1	M+H
D-Glyceraldehyde 3-phosphate	170.058	169.05	M-H
O-Acetyl-L-carnitine	203.236	204.1	M+H
Hypoxanthine	136.11	137	M+H
Acetylcarnitine	203.236	203.9	M+H
Trehalose 6-phosphate	422.276	421.076	M-H
1,4-Dihydro-1-Methyl-4-Oxo-3-Pyridinecarboxamide	152.151	153.06	M+H
2,4-Dihydroxybenzoic Acid	154.12	153	M-H
L-Octanoylcarnitine	287.395	288.3	M+H
Oxypurinol	152.11	153.04	M+H
DL-Citrulline	175.19	176.09	M+H
Carnitine-C8	287.3	288.3	M+H
Xanthine	152.11	151	M-H

L-Alanine	89.09	90	M+H
Adrenochrome	179.17	178.058	M-H
Hippuric acid	179.17	178.05	M-H
Taurochenodeoxycholic acid	499.7	498.2	M-H
D-3-Phenyllactic acid	166.17	165	M-H
Glucarate O-Phosphoric Acid	290.12	289.1	M-H
Vitamin B2	376.364	377.15	M+H
Hexanoylcarnitine	259.34	260.1	M+H
L-Homocystine	268.354	267.05	M-H
Carnitine-C6	259.3	260.2	M+H
Decanoylcarnitine	315.4	316.4	M+H
Dodecanoylcarnitine	343.501	344.4	M+H
Carnitine-C12	343.5	344.3	M+H
Isovalerylcarnitine	245.32	246.3	M+H
5-Aminosalicylate	153.135	154.05	M+H
Carnitine-C3	217.26	218.1	M+H
Carnitine-C5	245.32	246.1	M+H
Hydrocortisone	362.46	363.22	M+H
2-Methylbutyroylcarnitine	245.315	246.16	M+H
cholate	408.58	407.3	M-H
N-Propionylglycine	131.13	130.05	M-H
3-Oxo-7alpha,12alpha-hydroxy-5beta-cholanoic acid	406.562	405.2	M-H
Cinnamoylglycine	205.21	204.07	M-H
O-Acetyl-L-homoserine	161.16	162.1	M+H
L-Carnitine	161.2	162.1	M+H
Propionyl-L-carnitine	217.26	218.14	M+H
L-Alanyl-L-Lysine	217.265	218.1	M+H
Cortisone	360.44	361.112	M+H
Methylmalonate	118.09	117	M-H
3-Hydroxy-3-Methylpentane-1,5-Dioic Acid	162.14	163	M+H
DL-Norepinephrine	169.18	337.14	2M-H
Succinic acid	176.13	175.04	M-H
Nonanoic acid	158.24	159.1	M+H
3-OH-anthranilate	153.14	154	M+H
Deoxyguanosine	267.241	266.09	M-H
Uric Acid	168.11	167	M-H
Pantetheine	278.13	277.13	M-H
guanethidine	198.31	199.2	M+H
D-Glucosamine 6-phosphate	259.151	258.04	M-H
Indoxylsulfuric acid	213.21	212.01	M-H
CMPF	240.252	239.1	M-H
Hydroquinone	110.111	109.04	M-H

**p-cresol**	**108.14**	**107.058**	**M-H**
**3-Hydroxy-butyryl carnitine**	**247.29**	**248.3**	**M+H**
**Isocitrate**	**192.12**	**191.027**	**M-H**
**Tauroursodeoxycholic acid Dihydrate**	**499.7**	**498.2**	**M-H**
**4-Aminobenzoate**	**137.136**	**137.3**	**M+H**
**Tauro-alpha-Muricholic acid**	**515.7**	**514.2**	**M-H**
**Taurocholic acid**	**515.7**	**514.2**	**M-H**
**Furfural**	**96.08**	**97.021**	**M+H**
**O-Anisic Acid**	**152.147**	**153.05**	**M+H**
**Biotin**	**244.31**	**245**	**M+H**
**L-Glutamine O-Hexside**	**308.288**	**307**	**M-H**
**Pyridoxamine**	**168.193**	**168.9**	**M+H**
